# New Insight into Mechanisms of Cardiovascular Diseases: An Integrative Analysis Approach to Identify TheranoMiRNAs

**DOI:** 10.3390/ijms24076781

**Published:** 2023-04-05

**Authors:** Francesco Sessa, Monica Salerno, Massimiliano Esposito, Giuseppe Cocimano, Daniela Pisanelli, Abdul Malik, Azmat Ali Khan, Cristoforo Pomara

**Affiliations:** 1 Department of Medical, Surgical and Advanced Technologies “G.F. Ingrassia”, University of Catania, 95121 Catania, Italy; monica.salerno@unict.it (M.S.); massimiliano.esposito91@gmail.com (M.E.); cristoforo.pomara@unict.it (C.P.); 2 Department of Mental and Physical Health and Preventive Medicine, University of Campania “Vanvitelli”, 80121 Napoli, Italy; peppecocimano1@gmail.com; 3 Department of Clinical and Experimental Medicine, University of Foggia, 71100 Foggia, Italy; 4 Department of Pharmaceutics, College of Pharmacy, King Saud University, Riyadh 11451, Saudi Arabia; 5 Pharmaceutical Biotechnology Laboratory, Department of Pharmaceutical Chemistry, College of Pharmacy, King Saud University, Riyadh 11451, Saudi Arabia

**Keywords:** microRNAs (miRNAs), cardiovascular diseases (CVD), myocardial infarction, heart damage, heart failure, theranoMiRNAs

## Abstract

MiRNAs regulate both physiological and pathological heart functions. Altered expression of miRNAs is associated with cardiovascular diseases (CVDs), making miRNAs attractive therapeutic strategies for the diagnosis and treatment of heart diseases. A recent publication defined, for the first time, the term theranoMiRNA, meaning the miRNAs that may be used both for diagnosis and treatment. The use of in silico tools may be considered fundamental for these purposes, clarifying several molecular aspects, suggesting future directions for in vivo studies. This study aims to explore different bioinformatic tools in order to clarify miRNA interactions with candidate genes, demonstrating the need to use a computational approach when establishing the most probable associations between miRNAs and target genes. This study focused on the functions of miR-133a-3p, miR-21-5p, miR-499a-5p, miR-1-3p, and miR-126-3p, providing an up-to-date overview, and suggests future lines of research in the identification of theranoMiRNAs related to CVDs. Based on the results of the present study, we elucidated the molecular mechanisms that could be linked between miRNAs and CVDs, confirming that these miRNAs play an active role in the genesis and development of heart damage. Given that CVDs are the leading cause of death in the world, the identification of theranoMiRNAs is crucial, hence the need for a definition of in vivo studies in order to obtain further evidence in this challenging field of research.

## 1. Introduction

MiRNAs regulate both physiological and pathological heart functions, playing important epigenetic roles in gene regulation and cell function [1,2]. Altered expression of miRNAs is associated with heart metabolism dysregulation, heart injury, heart fibrosis, and, in general, cardiovascular diseases (CVDs), making miRNAs attractive therapeutic strategies for the diagnosis and treatment of heart diseases [3,4].

In the last few years, many articles have been published on this theme, confirming the growing interest in the identification of new molecular biomarkers [5,6]. A recent review analyzes the results of human studies over the last five years; according to the results of this review, the most-investigated miRNAs related to myocardial infarction (MI) and heart damage/failure are miR-133a-3p, miR-21, miR-499a-5p, miR-1, and miR-126. In the same paper, the term theranoMiRNA, meaning miRNAs that may be used both for diagnosis and treatment, was defined for the first time [7]. The identification of cardiac theranoMiRNAs remains a challenge for the scientific community.

**Figure 1:**
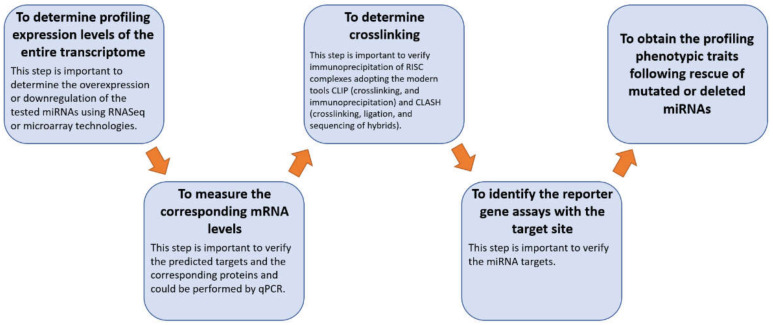
Diagram of the experimental protocols that could be applied to perform an in vivo study.

Experimental studies performed on human samples can provide direct links between miRNAs and their molecular targets; nevertheless, several important considerations should be taken into account. First of all, these studies are not error-free, and they require well-trained personnel; moreover, they are expensive (particularly when more than one miRNA is tested) in terms of both time and consumable costs [8,9,10]. The experimental protocols that could be applied to in vivo studies to analyze the miRNA targetome are summarized in Figure 1.

Moreover, these approaches should be used for different pathologies, linking the upregulation or downregulation of different miRNAs to pathology and vice-versa [11,12,13,14,15].

The use of in silico tools may be considered fundamental in this research field [16,17,18]. A recent article demonstrated the involvement of hsa-miR-124 and miR-16 in the biomolecular mechanisms of atherosclerosis, suggesting their potential use as diagnostic and therapeutic biomarkers (theranoMiRNAs) [19]. Another experimental study, using an integrated approach with an in silico analysis, demonstrated the involvement of different miRNAs in coronary artery disease (CAD) [20].

In this context, it seems clear that the molecular mechanisms underlying miRNA functions are not completely clear. Moreover, to date, in silico studies could be very useful to clarify several molecular aspects, suggesting future directions for in vivo studies [8,19,21,22,23]. This study aims to explore different bioinformatic tools in order to clarify miRNA interactions with candidate genes, showing the need to use a computational approach in establishing the most probable associations between miRNAs and target genes. In particular, this study focused on the functions of miR-133a-3p, miR-21-5p, miR-499a-5p, miR-1-3p, and miR-126-3p to provide an up-to-date overview and suggest future lines of research in the identification of theranoMiRNAs related to CVDs.

## 2. Results

### 2.1. miRBase Tool Analysis

The first step was the analysis of the sequence for each miRNA (Table 1).

This step is very important to allowing the repetition of the computational analysis.

**Figure 2:**
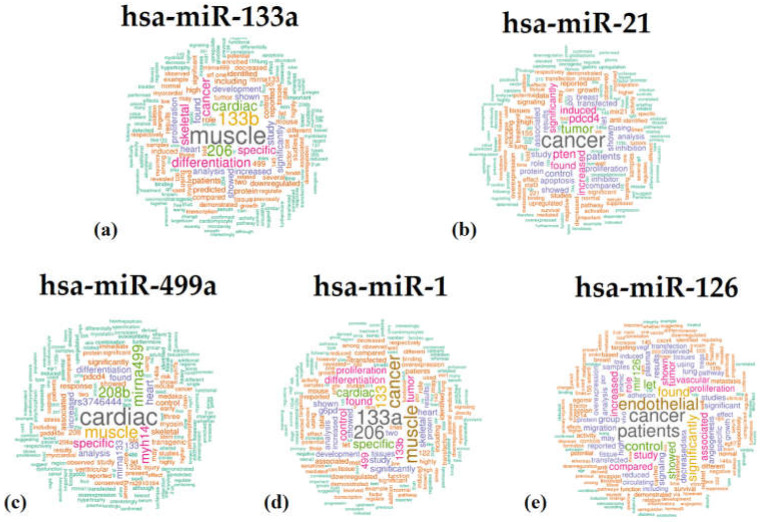
Word cloud for hsa-miR-133a (**a**); hsa-miR-21 (**b**); hsa-miR-499a (**c**); hsa-miR-1 (**d**); hsa-miR-126 (**e**).

Thanks to this tool, we obtained the following word cloud images (Figure 2) for each questioned miRNA.

Based on these graphic representations, hsa-miR-21-5p and hsa-miR-126-3p are more frequently related to the out-of-topic keywords, given that “cancer” was more frequent than “muscle/cardiac”, while the other miRNAs focused on the theme of this research article, matching with “cardiac”, “muscle”, or “miR-133a”.

Moreover, using the same tool, we checked the top 100 articles published about each miRNA, selecting—and summarizing in Table 2—the top 5 articles that focused on the CVD theme. For each article, we inserted the following information: rank (meaning the position within the 100 articles reported in the database), first author, the year, the title, the number of sentences that reported the name of the selected miRNA, and the other human miRNAs investigated (if only the miRNA of interest was investigated, then it is reported by the symbols “/“).

Analyzing the output, two studies were listed in the top 5 in the analysis of two miRNAs: Pisano et al. [26] is listed as the fourth study relative to the analysis of miR-133a-3p, and in the second position for the analysis of mir-499a-3p; Fu et al. [36] is listed in the top 5 articles in the investigations both on hsa-mir-499a and mir-1.

Based on the results obtained, the top 5 articles on the theme for hsa-miR-133a were listed in the first six positions. This supports the idea that this miRNA is strictly related to the modeling of cardiac functionality. On the contrary, the 5 top-ranking articles about miR-21 functionality have the worst rank, indicating that this miRNA is more involved in cancer regulation, as was also demonstrated in its word cloud.

### 2.2. Functional Annotation Analysis via the TAM 2.0 Tool

In the left panel of the TAM 2.0 tool, we inserted our miRNA list: hsa-miR-133a-3p; hsa-miR-21; hsa-miR-499a; hsa-miR-1; and hsa-miR-126.

**Figure 3:**
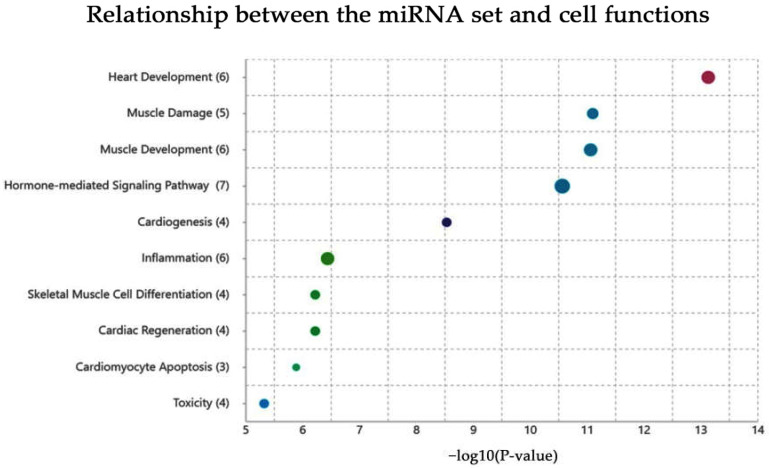
The relationship between the uploaded miRNA set and cell functions. As summarized in the graph, these miRNAs are involved in several essential functions, such as heart development, muscle damage, cardiogenesis, cardiac regeneration, and cardiomyocyte apoptosis.

Analyzing the results related to the tissue specificity category, this miRNA set resulted specific for heart development and muscle damages. In Figure 3, we have summarized the relationship between the uploaded miRNA set and cell functions.

**Figure 4:**
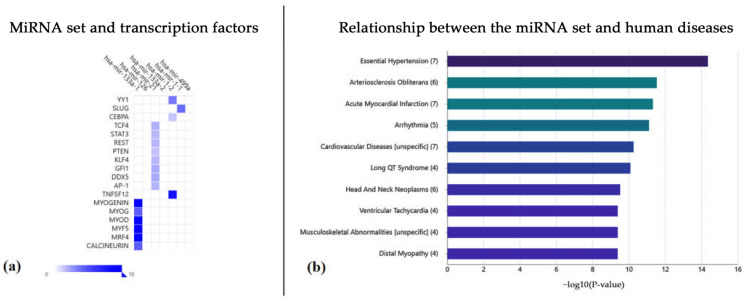
(**a**) Summary of the plot of the miRNA set and the transcriptional factors influenced by their molecular actions. (**b**) Summary of the main diseases correlated with this miRNA dataset.

Next, we analyzed the interaction of the questioned miRNA set and the transcription factors (Figure 4a), demonstrating a strict relationship with myogenin, MYOD (myoblast determination protein 1), MYF5 (myogenic factor 5), and MRF4 (also known as myogenic factor 6, MYF6) transcription factors. Myogenin is a transcriptional activator factor encoded by the MYOG gene [47,48,49]. This factor is muscle-specific (basic-helix-loop-helix, bHLH), and it is involved in the coordination of skeletal muscle development or myogenesis and repairment. Moreover, these miRNAs act on MYOD, an important protein that plays a pivotal role in muscle differentiation; in this way, it is important in CVD regulation. MYF5 is a human protein encoded by the MYF5 gene, and it plays a key role in regulating muscle differentiation or myogenesis, specifically in the development of skeletal muscle. Moreover, among the four myogenic regulatory factors (MRFs), MYF6 is the only factor that is primarily expressed in fully differentiated muscle fibers [50], and it has an important function promoting a vast set of myokines such as epidermal growth factor (EGF) and VEGFA (vascular endothelial growth factor A).

Based on these considerations, as summarized in Figure 4b, this miRNA set is involved in the regulation and control of several important diseases (such as essential hypertension, arteriosclerosis obliterans, acute myocardial infarction [AMI], arrhythmia, and long QT syndrome) that may cause CVDs.

### 2.3. miRTarBase Tool: Report about Experimentally Validated miRNA—Target Interactions of the Selected miRNAs

In order to ascertain the interaction between our miRNA set and the relative target, we used the miRTarBase tool.

The first analysis concerning the interactions experimentally validated was performed on hsa-miR-133a-3p. Based on the results of this tool, the main results pertaining to the interactions between this miRNA and the gene targets in human studies are summarized in Table 3.

**Figure 5:**
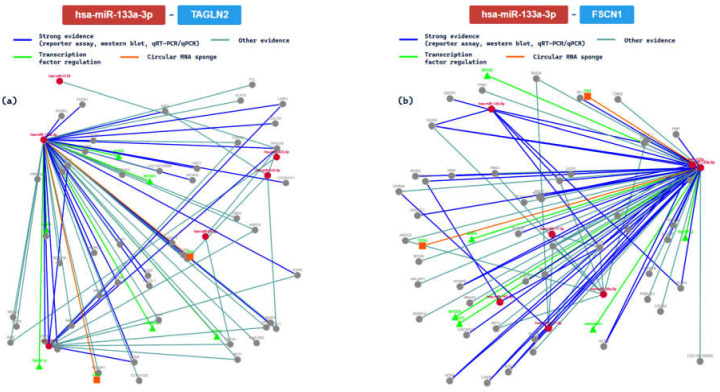
The regulatory network of has-miR-133a-3p and the TAGLN2 gene (**a**); (**b**) the regulatory network of interaction between the tested miRNA and FSCN1 gene.

Moreover, we further analyzed the interaction with 5 methods. Specifically, we analyzed the network interactions with the gene targets TAGLN2 (transgelin 2) and FSCN1 (fascin actin-bundling protein 1), summarizing them in Figure 5.

The TAGLN2 gene encoded for a protein similar to transgelin, which is one of the earliest markers of differentiated smooth muscle [51]. Based on the results of the gene ontology analysis performed with the same tool, this miRNA actively acts on the TAGLN2 gene, influencing different processes involved in the modulation of the frequency rate or of heart contraction [52]. Heart contraction is a process in which the heart decreases in volume in a characteristic way in order to propel blood through the body, and it is mediated by hormonal, autocrine, or paracrine chemical signaling. The FSCN1 gene encodes a member of the fascin family of actin-binding proteins [53]. The interactions between hsa-miR-133a and this gene are important, because they are involved in the circulatory system process carried out by the heart, influencing the muscular organ, controlling the rhythm, and influencing the cardiac conduction process that modulates the frequency or rate of heart contraction.

We then analyzed the output related to the analysis of hsa-miR-21. Based on the analysis performed, we obtained several gene targets in human models. The main results are summarized in Table 4.

**Figure 6:**
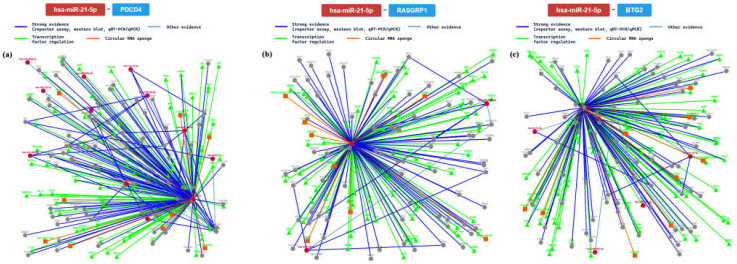
The regulatory network of has-miR-21 and the PDCD gene (**a**); (**b**) shows the regulatory network of interaction between the tested miRNA and the RASGRP1 gene; (**c**) shows the network relative to the BTG2 gene.

Additionally, we further analyzed gene interactions that had been confirmed with at least 6 methods. In this way, we analyzed the interaction network among miR-21 and PDCD4 (programmed cell death 4, validated with 7 methods), RASGRP1 (RAS guanyl-releasing protein 1, validated with 6 methods), and BTG2 (B-cell translocation gene 2, validated with 6 methods) genes. The main results are summarized in Figure 6.

The PDCD4 gene is involved in important processes in cell regulation, considering that it is a tumor suppressor. It encodes a protein that binds to the eukaryotic translation initiation factor 4 A1 and inhibits its function by preventing RNA binding [54]. Based on gene ontology analysis, it is involved in heart development, considering its role in epithelial to mesenchymal transition. Specifically, it plays an important role in the delineation of a specific region of the lateral mesoderm into the area which will form the primary beating heart tube. In this scenario, it may be directly involved in CVD genesis. The RASGRP1 gene is a member of the Ras superfamily guanine nucleotide exchange factor (GEF) domain. It exerts its function on a diacylglycerol (DAG)-regulated nucleotide exchange factor, acting on Ras through the exchange of bound GDP for GTP [55]. In this way, it may activate the Erk/MAP kinase cascade, regulating T-cell and B-cell development, homeostasis, and differentiation. Altered protein expression of the encoded protein may be a cause of susceptibility to systemic lupus erythematosus (SLE). This gene is involved in heart morphogenesis, influencing the developmental process. Moreover, it plays an important role in the regulation of heart valve morphogenesis. The BTG2 gene is a member of the BTG/Tob family. The related proteins may have antiproliferative properties. Moreover, the protein encoded by this gene is involved in the regulation of the G1/S transition of the cell cycle [56]. Considering heart development, the BTG2 gene is involved in the tube morphogenesis process in which the primitive heart tube loops asymmetrically. This looping brings the primitive heart chambers into alignment, preceding their future integration. For these reasons, this gene may be involved in different CVDs.

Analyzing hsa-miR-499a-5p molecular interaction with this tool for the papers focused on human models, we obtained the results summarized in Table 5.

**Figure 7:**
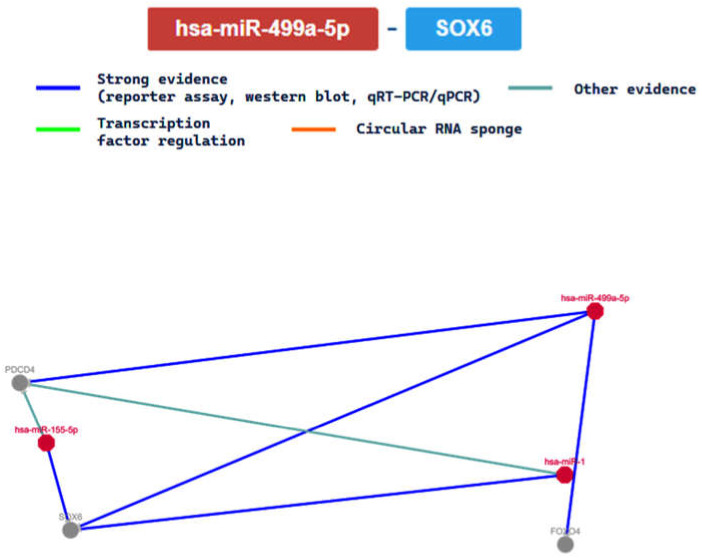
The regulatory network of hsa-miR-499a-5p and the SOX6 gene.

Moreover, we further analyzed the interaction between hsa-miR-499a-5p and the SOX6 (SRY-box transcription factor 6) gene, confirmed with 4 methods, reporting the interaction network in Figure 7. As demonstrated in the same figure, the network is easily compared to others, this meaning that its relationship seems to be clear.

The SOX6 gene is considered a tumor suppressor gene, and it is a member of the D subfamily of sex-determining region y-related transcription factors. It plays important roles, considering that its encoded protein represents a transcriptional activator involved in the normal development of the central nervous system, chondrogenesis, and maintenance of cardiac and skeletal muscle cells [57]. Based on gene ontology, it is involved in the process of vasculature over time, from its formation to the mature structure. In terms of its role in the circulatory system it is actively involved in several important processes, such as passing nutrients (i.e., amino acids and electrolytes), gases, hormones, blood cells, etc., to and from cells in the body in order to help fight diseases and help stabilize body temperature and pH so as to maintain homeostasis.

Analyzing an hsa-miR-1-3p model with this tool, and analyzing the papers published concerning human studies, we obtained the results summarized in Table 6.

**Figure 8:**
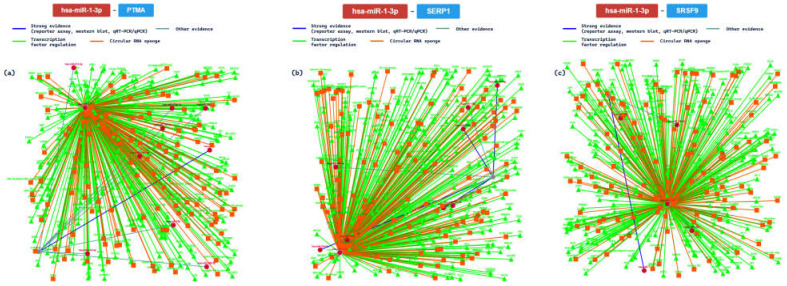
The regulatory network of has-miR-1 and the PTMA gene (**a**); (**b**) shows the regulatory network of the interaction between the tested miRNA and the SERP1 gene; (**c**) shows the network relative to the SRSF9 gene.

Additionally, we further analyzed the interaction confirmed with at least 7 methods, analyzing the interaction network among miR-1 and PTMA (prothymosin alpha, validated with 8/8 methods), SERP1 (stress-associated endoplasmic reticulum protein 1, 7/8), and SRSF9 (serine and arginine-rich splicing factor 9, 7/8) genes. The main results are summarized in Figure 8.

Thanks to the graphical representation, it is clear that the interactions based on strong evidence (blue line) represent a minor portion of the networks. The PTMA gene encoded for a protein involved in complex pathways such as validated targets of C-MYC transcriptional activation and the VEGFA–VEGFR2 signaling pathway. This is very important, because it may mediate immune function by providing resistance to certain opportunistic infections [58]. At the cardiac level, it is involved in the regulation of several important functions, including heart development and heart contraction (regulation of heart rate by cardiac conduction). The SERP1 gene encodes a protein that interacts with target proteins during their translocation into the lumen of the endoplasmic reticulum. Moreover, this protein exerts an important function, protecting unfolded target proteins against degradation during ER stress. Additionally, it can facilitate the glycosylation of target proteins, and it may modulate the use of N-glycosylation sites on target proteins [59]. At the heart level, it may regulate the cell migration and the cardiac neural crest cell differentiation involved in heart development. The SRSF9 gene encodes a protein that plays a role in constitutive splicing and can modulate the selection of alternative splice sites. Specifically, it represses the splicing of MAPT/Tau exon 10 [60]. At cardiac levels, it may modulate the frequency, rate, or extent of heart contraction: in this way, this protein is important in determining heart functionality.

Finally, we analyzed and experimentally validated the interactions with hsa-miR-126-3p. Based on the results of the used tool, the main results concerning the interactions between this miRNA and the gene targets in human studies are summarized in Table 7.

**Figure 9:**
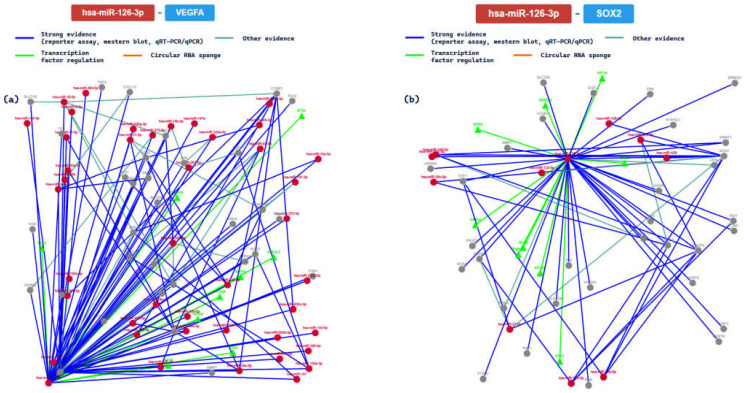
The regulatory network of has-miR-126 and the VEGFA gene (**a**); (**b**) the regulatory network of interaction between the tested miRNA and the SOX2 gene.

We further analyzed the interactions between the miRNA and the relative genes for those confirmed with at least 6 methods. In this way, we analyzed the interaction network among miR-126 and VEGFA (7/8 validation methods) and the SOX2 (SRY-box transcription factor 2, 7/8) genes. The main results are summarized in Figure 9.

The VEGFA gene is a member of the PDGF/VEGF growth factor family. It plays essential roles in physiological and pathological angiogenesis, and it has been reported to be upregulated in many known tumors. It has been reported to play an important role in inflammation: angiopoietin II (Ang II) facilitates the elevation of VEGF, releasing inflammatory cytokines [61]. It is important in mediating heart inflammation and heart development. Moreover, it interacts with different miRNAs both to promote and inhibit different molecular functions. The SOX2 gene encodes a protein of the SRY-related HMG-box (SOX) family, a family of transcription factors involved in the regulation of embryonic development and in the determination of cell fate [62]. At the cardiac level, it promotes several important functions in order to maintain homeostasis and heart functionality.

## 3. Discussion

It has now been established that miRNAs are biomolecules involved in almost all biological processes of CVDs. Based on in silico analysis, our set of miRNAs can be considered important in the genesis of CVDs. Moreover, the same miRNAs may represent a therapeutic target for preventing the insurgence of CVDs.

Interestingly, the use of prediction tools has exploded in the last few decades using different approaches. For example, Mukushkina et al. [63] investigated the involvement of miRNAs in the development of atherosclerosis, trying to clarify the characteristics of interactions of miRNAs with mRNA candidate genes. Using different tools, the authors concluded that different miRNAs could be used for the early diagnosis of this disease. In this regard, Kern et al. [64] proposed a hierarchical clustering of 98 target prediction methods based on 16 categorical variables illustrated as a polar tree dendrogram. This approach could be very useful in choosing the appropriate approach for computational studies.

Given that in experimental studies it would be desirable to have lower costs for disease diagnosis and treatment, it would be beneficial to minimize the number of associations between miRNAs and target genes. However, in the case of polygenic diseases such as those related to CVDs, the establishment of well set-up experimental designs may be difficult, because it is not known which gene and which miRNA may be linked with the development of the disease [65,66]. Therefore, it would be desirable to define by in silico analysis a list of priority theranostic genes that can then be studied by different techniques in order to identify theranoMiRNAs that may influence protein expression.

Based on the results proposed through this in silico study, these miRNAs could potentially be used in clinical applications, such as in drug development and personalized medicine. As recently described, bioinformatic analysis is the first step in the definition of new drug treatments, considering that the FDA has approved the use of small RNA drugs. In this way, in the last few years, preclinical and clinical research studies have grown exponentially. The following step should be the in vitro *and* in vivo validation that is essential for clinical research studies such as those on medical intervention drugs [67]. This last step is generally the most expensive both in terms of time and resources; despite this though, the identification of theranoMiRNAs related to CVDs remains an important goal for the scientific community. Moreover, interest in personalized medicine has considerably increased in the last few years [68,69], and it is important to remark that this area of medicine is based on detecting and evaluating the characteristics of each patient. For this reason, the use of theranoMiRNAs could be considered the future of medicine, because it could be tailored to each individual following the idea of personalized medicine [70].

Thanks to in silico tools, the adoption of a computational prediction of canonical or non-canonical miRNA targets, and the application of different statistical and machine-learning approaches, it is possible to determine the degree of pairing between the miRNA seed region and target site; the conservation degree across species; the thermodynamic properties; the accessibility of target sites; and the sequence composition both of miRNA and target sites [71,72,73,74].

As reported in a recent experimental article performed on an in vitro model, the biochemical mechanism of miR-133a seems to be related to angiotensin II action, inducing cardiac hypertrophy [75]. Moreover, it has been reported that it could be involved in atherosclerosis progression, acting through lipid and inflammatory signaling [76]. Based on a recent review, this miRNA is involved in different CVDs, given that it was downregulated in cardiac hypertrophy and Long Q-T Syndrome (LQTS), while it is upregulated in arrhythmias. Finally, analyzing the data relative to myocardial infarction, the results are in contrast, as several reports described that it is upregulated and others that it is downregulated [77]. The in silico analysis performed in this study confirmed these findings: miR-133a actively acts on TAGLN2 and FSCN1 genes, regulating different processes involved in the modulation of the frequency rate or heart contraction. These results support the idea that this miRNA is strictly related to the modeling of cardiac functionality, and it may be considered a theranoMiRNA.

In terms of the role of has-miR-21 in the progression of cardiac damage (such as dilated cardiomyopathy, myocardial infarction, and diabetic cardiomyopathy), it could be involved in the regulation of different cardiac functions, targeting different mRNAs [78,79]. Additionally, it has been conjectured that it may promote transforming growth factor-beta 1 (TGF-β1)-induced fibroblast activation, increasing the expression of Collagen-1, alpha-smooth muscle actin (α-SMA), and F-actin [79]. A recent study, based on an integrated approach with in silico analysis, confirmed a pivotal role for has-miR-21 in CAD [20]. Moreover, is has been reported that this miRNA is upregulated in arrhythmogenic cardiomyopathy (ACM) and dilated cardiomyopathy (DCM), two inherited cardiomyopathies [77]. Based on the results of the present study, the role of miR-21 in CVDs is related to its interaction with the following genes: PDCD4, RASGRP1, and BTG2. Particularly, this study demonstrated that, through the interactions with the reported genes, it plays a pivotal role in the regulation of important molecular functions, confirming that it should be considered an ideal candidate both for diagnosis and therapy.

Based on recent studies, has-miR-499a-5p seems to be involved in the prevention of doxorubicin-induced cardiotoxicity, targeting p21 and reducing mitochondrial fission and apoptosis [80]. In this manner, it seems to be involved in cardiomyocyte damage following hypoxia/reoxygenation (H/R) [81,82]. A recent review reported the involvement of this miRNA in ACM, resulting in downregulation [77]. The present study demonstrated that this miRNA plays acts on the SOX6 gene, regulating important processes in cardiac cells, and, consequently, it is involved in the insurgence and evolution of CVDs.

Considering the molecular mechanism behind has-miR-1 regulation, it has been reported that miR-1 is differently expressed in two cardiac injuries—hypertrophic cardiomyopathy (HCM) and DCM—allowing for differentiation between them. In particular, it seems to be involved in the regulation of cardiac contractile function, despite the fact that the molecular interactions are not fully explained [83,84]. Colpaert and Calore reported that it is upregulated in contractility defects and arrhythmias, while it seems to be downregulated in cardiac hypertrophy. Interestingly, it is downregulated in two inherited cardiomyopathies (DCM, and LQTS) [77]. Based on this in silico analysis, MiR-1 is involved in the CVDs, acting on the PTMA, SERP1, and SRSF9 genes and regulating different pivotal mechanisms such as cardiac neural crest cell differentiation involved in heart development; a molecular alteration of these pathways represents the cause of different CVDs.

Finally, has-miR-126 has been reported to be a biomarker of cardiovascular disease (CVD), considering that this miRNA is differently expressed in patients with HF compared with healthy controls. It acts by impacting several mRNAs involved in the inflammatory cascade [85,86]. Based on the results of in silico analysis, this miRNA is involved in the regulatory post-transcriptional factors of the VEGFA and SOX2 genes. As previously discussed, it plays a pivotal role in pathophysiological angiogenesis, and it may be involved in CVD insurgence.

As has largely been discussed, miR-133a, miR-21, miR-499a, miR-1, and miR-126 may be considered ideal theranomiRNA candidates for CVDs.

This study has several strengths: first of all, it has investigated different tools in order to obtain the best performance for each one. Moreover, starting from the miRNA sequence, it has analyzed different aspects such as cell functions, the relationships to both transcriptional factors and human diseases, and finally the gene target and miRNA functionality. At the same time, this study has several limitations: if, on the one hand, the use of a computational approach could be considered important in the pre-evaluation of miRNA function, on the other hand, it should be taken into account that it should be confirmed with in vivo experimental studies. Moreover, the results of the present study are strictly related to the last version of each used tool: in the near future, the use of new release versions of the same tools, as well as the release of new tools, could change the present results.

## 4. Materials and Methods

In the present study, we investigated miRNAs based on the results of a recent literature review that identified the most-investigated miRNAs in human models related to CVDs. In particular, this study focused on the functions of hsa-miR-133a, hsa-miR-21, hsa-miR-499a, hsa-miR-1, and hsa-miR-126.

**Figure 10:**
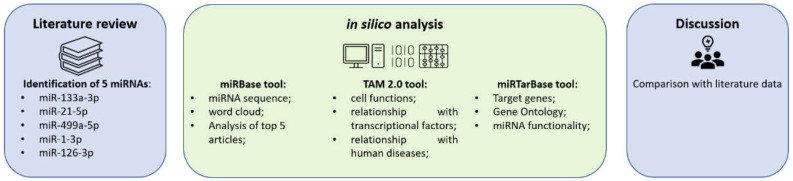
The workflow adopted to perform this in silico study.

The methodologies and strategies used in this paper are summarized in Figure 10.

### 4.1. miRNA Selection

In accordance with a recent systematic review that focused on the miRNAs investigated in human studies in the last 5 years, this in silico study focused on the role of hsa-miR-133a, hsa-miR-21, hsa-miR-499a, hsa-miR-1, and hsa-miR-126 in CVDs. Indeed, out of the 59 selected articles, miR-133a-3p had been investigated 10 times, miR-21 and miR-499a-5p were investigated 8 times each, miR-1 was studied 6 times, and miR-126 was investigated 5 times [7].

Moreover, based on the same data, these miRNAs could be used both for the diagnosis and for the treatment of CVDs, opening the idea of defining them with the new term, coined for the first time in the same article: “TheranoMiRNAs” [7].

### 4.2. miRBase Tool

miRbase (available at the following link https://www.mirbase.org/, accessed on 4 February 2023) is a searchable database of published miRNA sequences and annotations; the current version used for this paper is 22.1, based on 38,589 entries [87]. For each miRNA entry, the miRBase database predicted a sequence database, representing a hairpin portion of a miRNA transcript, giving information on the location and sequence of the mature miRNA sequence (termed miR). For this study, we retrieved the miRNA sequence and the relative “Word cloud” obtained for each miRNA. Moreover, given that this database contains a link with the top 100 articles retrieved from PubMed, we selected the top 5 articles in order of relevance, selecting among these those that investigated cardiac function. This function is specific to this tool and has been fully described by Kozomara et al. [87].

### 4.3. TAM 2.0 Tool

In order to analyze the functional annotation of the selected miRNAs, we used the TAM 2.0 tool [88]. This tool (available at the following link: http://www.lirmed.com/tam2/, accessed on 4 February 2023) is free for academic usage, and it is based on the analysis of about 9 k papers, allowing for the determination of each miRNA set’s association with different diseases, miRNA families, transcription factors, and biological functions.

Summarizing the main functionality, it allows for the upload of the miRNA list, choosing between overrepresentation and underrepresentation, enabling the possibility to select between up- (or disease promotion) and downregulation (or disease suppression).

Based on its data setting, when a researcher inserts the mature miRNA names (e.g., hsa-miR-133a-3p), the name will be collapsed into the corresponding miRNA gene (hsa-mir-133a). On the contrary, if the exact name of the duplicated miRNA gene is not provided, the software will analyze all of the duplicated miRNA genes.

We used this tool to obtain the following information for the analyzed miRNA set: the cell functions, the relationship with transcriptional factors, and related human diseases.

### 4.4. miRTarBase Tool

miRTarBase is a database that provides data on miRNA–target interactions verified by biological experiments. The data in this database are subject to periodic revision. The version used for this work is Release 9.0 beta (dated 15 September 2021, available at https://miRTarBase.cuhk.edu.cn/, accessed on 4 February 2023), which includes data obtained from 13,398 articles involving 37 species. The number of target genes is 27,172, while the number of miRNAs is 4630, identifying 2,200,449 miRNA–target interactions [89]. Thanks to this tool, we analyzed each tested miRNA in order to define the target genes and, through the gene ontology, miRNA functionality.

## 5. Conclusions

The definition of new theranoMiRNAs for CVDs, as a means to identify new molecular markers that can be used for both diagnostic and therapeutic purposes, represents an ambitious goal for the scientific community. Despite the enormous scientific output of miRNAs for the diagnosis and prognosis of CVDs, to date, our knowledge in this field can still be considered superficial. To clarify the full potential of miRNAs, more robust sample collection processing and advances in technologies and analytical methods are necessary.

In our opinion, rather than the discovery of new miRNAs, it could be important to confirm already-published results by trying to create large research networks, involving industry and experts in fields as diverse as molecular biology, analytical chemistry, bioinformatics, clinical trial design, epidemiology, statistics, and health economics. In a historical context where, on the one hand, the development of new technologies takes place daily, on the other hand, the budget reserved for research is increasingly limited, the use of computational studies applied to clinical research has become one of the cornerstones to be considered indispensable. In particular, computational studies are crucial in setting up well-designed scientific studies, which can lead, on the one hand, to the optimization of costs and investigation time, and, on the other hand, to obtaining scientifically valid results. In this study, we analyzed the molecular mechanisms that could be linked between our miRNAs set and CVDs, confirming that the selected miRNAs, hsa-miR-133a, hsa-miR-21, hsa-miR-499a, hsa-miR-1, and hsa-miR-126, play an active role in the genesis and development of different heart damage.

Given that CVDs are the leading cause of death in the world, the identification of theranoMiRNAs is crucial, hence the need for a definition of well-designed in vivo studies in order to obtain further evidence in this challenging field of research.

## Figures and Tables

**Table 1:** Summary of the main information of the tested miRNAs, the accession number inserted into this tool, the sequence of the mature miRNA, and the genomic localization.

miRNA (Mature Sequence)	Accession Number	Sequence	Genomic Localization
hsa-miR-133a-3p	MIMAT0000427	UUUGGUCCCCUUCAACCAGCUG	chr18: 21825698-21825785 [−]
hsa-miR-21-5p	MIMAT0000076	UAGCUUAUCAGACUGAUGUUGA	chr17: 59841266-59841337 [+]
hsa-miR-499a-5p	MIMAT0002870	UUAAGACUUGCAGUGAUGUUU	chr20: 34990376-34990497 [+]
hsa-miR-1-3p	MIMAT0000416	UGGAAUGUAAAGAAGUAUGUAU	chr18: 21829004-21829088 [−]
hsa-miR-126-3p	MIMAT0000445	UCGUACCGUGAGUAAUAAUGCG	chr9: 136670602-136670686 [+]

**Table 2:** The top 5 selected, ranked articles on the theme of this article for hsa-miR-133a-3p, hsa-miR-21-5p, hsa-miR-499a-5p, hsa-miR-1-3p, and hsa-miR-126-3p; additionally, the number of sentences associated with the microRNA’s name, and other human microRNAs that were screened in the study have been inserted.

	Rank, First Authors, Year	Article Title	N° of Sentences	Other Human miRNAs
hsa-miR-133a-	n° 1, Ohanian et al., 2013 [24]	A heterozygous variant in the human cardiac miR-133 gene, MIR133A2, alters miRNA duplex processing and strand abundance.	85	miR-96, miR-1-2, miR-133a-2, miR-206, miR-1-1, miR-133b, miR-499a, miR-499b
	n° 3, Nielsen et al., 2013 [25]	Muscle specific miRNAs are induced by testosterone and independently upregulated by age.	48	miR-1-2, miR-133a-2, miR-206, miR-1-1, miR-133b
	n° 4, Pisano et al., 2015 [26]	Combination of miRNA499 and miRNA133 exerts a synergic effect on cardiac differentiation.	60	miR-1-2, miR-133a-2, miR-1-1, miR-133b, miR-449a, miR-499a, miR-449b, miR-449c, miR-499b
	n° 5, Deng et al., 2011 [27]	Transgenic overexpression of miR-133a in skeletal muscle.	53	miR-208a, miR-214, miR-221, miR-222, miR-1-2, miR-133a-2, miR-206, miR-1-1, miR-155, miR-133b, miR-146b, miR-499a, miR-208b, miR-499b
	n° 6, Pahl et al., 2012 [28]	MicroRNA expression signature in human abdominal aortic aneurysms.	27	miR-21, miR-29b-1, miR-29b-2, miR-30c-2, miR-181a-2, miR-204, miR-211, miR-181a-1, miR-133a-2, miR-146a, miR-331, miR-133b, miR-146b
hsa-miR-21	n° 7, Chen et al., 2013 [29]	4-HNE increases intracellular ADMA levels in cultured HUVECs: evidence for miR-21-dependent mechanisms.	97	/
	n° 8, Liu et al., 2011 [30]	MiR-21 induced angiogenesis through AKT and ERK activation and HIF-1α expression.	81	let-7a-1, let-7a-2, let-7a-3, let-7b, let-7c, let-7d, let-7e, let-7f-1, let-7f-2, let-7g, let-7i, miR-27b, miR-130a, miR-126, miR-296, miR-378a, miR-378d-2, miR-378b, miR-378c, miR-378d-1, miR-378e, miR-378f, miR-378g, miR-378h, miR-378i, miR-378j
	n° 9, Song et al., 2012 [31]	Mechanical stretch modulates microRNA 21 expression, participating in proliferation and apoptosis in cultured human aortic smooth muscle cells.	93	miR-19a, miR-26a-1, miR-23b, miR-26a-2
	n° 12, Zhu et al., 2014 [32]	MicroRNA-21 regulates hTERT via PTEN in hypertrophic scar fibroblasts.	103	/
	n° 60, Green et al., 2014 [33]	PPARγ Ligands Attenuate Hypoxia-Induced Proliferation in Human Pulmonary Artery Smooth Muscle Cells through Modulation of MicroRNA-21.	71	miR-204
hsa-miR-499a	n° 1, Shieh et al., 2011 [34]	Elevated miR-499 levels blunt the cardiac stress response.	107	miR-16-1, miR-16-2, miR-208a, miR-1-2, miR-133a-1, miR-133a-2, miR-206, miR-1-1, miR-133b, miR-208b, miR-499b
	n° 2, Pisano et al., 2015 [26]	Combination of miRNA499 and miRNA133 exerts a synergic effect on cardiac differentiation.	60	miR-1-2, miR-133a-2, mirR-1-1, miR-133b, miR-449a, miR-499a, miR-449b, miR-449c, miR-499b
	n° 3, Bhuiyan et al., 2013 [35]	Evolution of the myosin heavy chain gene MYH14 and its intronic microRNA miR-499: muscle-specific miR-499 expression persists in the absence of the ancestral host gene.	95	miR-499b
	n° 6, Fu et al., 2013 [36]	Distinct roles of microRNA-1 and -499 in ventricular specification and functional maturation of human embryonic stem cell-derived cardiomyocytes.	42	let-7a-1, let-7a-2, let-7a-3, let-7b, let-7c, let-7d, let-7e, let-7f-1, let-7f-2, miR-21, miR-26b, miR-27a, miR-30a, miR-208a, let-7i, miR-1-2, miR-27b, miR-30b, miR-133a-1, miR-133a-2, miR-143, miR-125a, miR-126, miR-188, miR-1-1, miR-302a, miR-296, miR-302b, miR-302c, miR-302d, miR-371a, miR-133b, miR-208b, miR-302e, miR-302f, miR-371b, miR-499b
	n° 7, Wang et al., 2012 [37]	Gadd45α: a novel diabetes-associated gene potentially linking diabetic cardiomyopathy and baroreflex dysfunction.	29	miR-1-2, miR-133a-1, miR-133a-2, miR-320a, miR-1-1, miR-320b-1, miR-320c-1, miR-320b-2, miR-320d-1, miR-320c-2, miR-320d-2, miR-320e, miR-499b
hsa-miR-1	n° 5, Fu et al., 2013 [36]	Distinct roles of microRNA-1 and -499 in ventricular specification and functional maturation of human embryonic stem cell-derived cardiomyocytes.	42	let-7a-1, let-7a-2, let-7a-3, let-7b, let-7c, let-7d, let-7e, let-7f-1, let-7f-2, miR-21, miR-26b, miR-27a, miR-30a, miR-208a, let-7i, miR-1-2, miR-27b, miR-30b, miR-133a-1, miR-133a-2, miR-143, miR-125a, miR-126, miR-188, mir-1-1, miR-302a, miR-296, miR-302b, miR-302c, miR-302d, miR-371a, miR-133b, miR-208b, miR-302e, miR-302f, miR-371b, miR-499b
	n° 7, Zhang et al., 2011 [38]	Regulation of cardiac microRNAs by serum response factor.	31	miR-21, miR-199a-1, miR-199a-2, miR-1-2, miR-133a-1, miR-133a-2, miR-381, miR-499a, miR-499b
	n° 11, Li et al., 2013 [39]	An intragenic SRF-dependent regulatory motif directs cardiac-specific microRNA-1-1/133a-2 expression.	37	miR-1-2, miR-133a-1, miR-133a-2, miR-133b
	n° 12, Ceci et al., 2018 [40]	Micro RNAs are involved in activation of epicardium during zebrafish heart regeneration.	24	miR-1-2, miR-133a-1, miR-133a-2, miR-133b
	n° 13, Koutsoulidou et al., 2018 [41]	Expression of miR-1, miR-133a, miR-133b and miR-206 increases during development of human skeletal muscle.	28	miR-1-2, miR-133a-1, miR-133a-2, miR-206, miR-133b
hsa-miR-126	n° 2, Witkowski et al., 2016 [42]	Micro-RNA-126 Reduces the Blood Thrombogenicity in Diabetes Mellitus via Targeting of Tissue Factor.	105	miR-19a, miR-19b-1, miR-19b-2
	n° 5, Qin et al., 2013 [43]	MicroRNA-126 regulates the induction and function of CD4+ Foxp3+ regulatory T cells through PI3K/AKT pathway	97	miR-15a, miR-15b, miR-146a, miR-155
	n° 10, Wang et al., 2016 [44]	miR-126 Regulation of Angiogenesis in Age-Related Macular Degeneration in CNV Mouse Model.	70	miR-23a, miR-31, miR-23b, miR-122, miR-145, miR-146a, miR-150, miR-184, miR-23c
	n° 11, Mondadori et al., 2015 [45]	miR-126 Is Involved in Vascular Remodeling under Laminar Shear Stress.	59	miR-222
	n° 12, Izuhara et al., 2015 [46]	Prevention of neointimal formation using miRNA-126-containing nanoparticle-conjugated stents in a rabbit model.	89	/

**Table 3:** Summary of the miRNA interactions referred to hsa-miR-133a-3p, supported with at least 4 positive tools; green = validated methods, red = unvalidated methods. The methods are distinguished as strong or less-strong tools.

miRTarBase ID	Target	Validation Methods							
		Strong Evidence			Less-Strong Evidence				
		Reported Assay	Western Blot	qPCR	Microarray	NGS	pSILAC	Other	CLIP-Seq
MIRT005604	TAGLN2								
MIRT003542	FSCN1								
MIRT000327	CDC42								
MIRT001204	HCN2								
MIRT002925	KCNQ1								
MIRT004831	KCNH2								
MIRT006571	PNP								
MIRT021705	ARPC5								
MIRT021714	COL1A1								
MIRT052647	BCL2L1								
MIRT052648	MCL1								
MIRT053333	RFFL								
MIRT081957	UBA2								
MIRT437953	ANXA2								
MIRT437954	SNX30								
MIRT437955	SGMS2								
MIRT737550	TGFB1								

**Table 4:** Summary of the miRNA interactions referred to hsa-miR-21, supported with at least 6 positive tools; green = validated methods, red = unvalidated methods. The methods have been distinguished as strong or less-strong tools.

miRTarBase ID	Target	Validation Methods							
		Strong Evidence			Less-Strong Evidence				
		Reported Assay	Western Blot	qPCR	Microarray	NGS	pSILAC	Other	CLIP-Seq
MIRT003054	PDCD4								
MIRT000019	RASGRP1								
MIRT002416	BTG2								
MIRT000157	CDC25A								
MIRT000159	BCL2								
MIRT000168	RPS7								
MIRT000954	TIMP3								
MIRT000960	SOX5								
MIRT000969	RECK								
MIRT001189	TGFBR2								
MIRT001190	PTEN								
MIRT001980	TPM1								
MIRT002406	CDK6								
MIRT002410	APAF1								
MIRT003567	SERPINB5								
MIRT003837	BMPR2								
MIRT005429	MSH2								
MIRT005807	EGFR								

**Table 5:** Summary of the miRNA interactions referred to hsa-miR-499a-5p, supported with at least 3 positive tools; green = validated methods, red = unvalidated methods. The methods have been distinguished as strong or less-strong tools.

miRTarBase ID	Target	Validation Methods							
		Strong Evidence			Less-Strong Evidence				
		Reported Assay	Western Blot	qPCR	Microarray	NGS	pSILAC	Other	CLIP-Seq
MIRT004658	SOX6								
MIRT006698	FOXO4								
MIRT006699	PDCD4								
MIRT054151	ETS1								
MIRT734866	VAV3								

**Table 6:** Summary of the miRNA interactions referred to hsa-miR-1-3p, supported with at least 5 positive methods; green = validated methods, red = unvalidated methods. The methods have been distinguished as strong or less-strong tools.

miRTarBase ID	Target	Validation Methods							
		Strong Evidence			Less-Strong Evidence				
		Reported Assay	Western Blot	qPCR	Microarray	NGS	pSILAC	Other	CLIP-Seq
MIRT001343	PTMA								
MIRT002806	SERP1								
MIRT005072	SRSF9								
MIRT001357	MET								
MIRT002788	TAGLN2								
MIRT002956	G6PD								
MIRT004670	PAX3								
MIRT005089	TWF1								
MIRT001364	GPD2								
MIRT000933	HCN4								
MIRT001375	CORO1C								
MIRT001388	ANXA2								
MIRT001983	KCNJ2								
MIRT002751	XPO6								
MIRT002808	LASP1								
MIRT003765	PNP								

**Table 7:** Summary of the miRNA interactions with hsa-miR-126-3p, supported with at least 5 positive tools: green = validated methods, red = unvalidated methods. The methods have been distinguished as strong or less-strong tools.

miRTarBase ID	Target	Validation Methods							
		Strong Evidence			Less-Strong Evidence				
		Reported Assay	Western Blot	qPCR	Microarray	NGS	pSILAC	Other	CLIP-Seq
MIRT003428	VEGFA								
MIRT005370	SOX2								
MIRT000967	TOM1								
MIRT002993	CRK								
MIRT005538	TWF1								
MIRT006679	SLC7A5								

## Data Availability

All data are included in this manuscript.
